# AI-assisted identification of nonmelanoma skin cancer structures based on combined line-field confocal optical coherence tomography and confocal Raman microspectroscopy

**DOI:** 10.1117/1.JBO.30.7.076008

**Published:** 2025-07-28

**Authors:** Meriem Ayadh, Léna Waszczuk, Jonas Ogien, Grégoire Dauce, Luc Augis, Sana Tfaili, Ali Tfayli, Jean-Luc Perrot, Arnaud Dubois

**Affiliations:** a Dermatologie CHU Saint-Etienne, Laboratoire de Tribologie et Dynamique des Systèmes UMR CNRS 5513, Saint-Etienne, France; b Damae Medical, Paris, France; c Lip(sys)2, Chimie Analytique Pharmaceutique, Laboratoire de Chimie Analytique Pharmaceutique, Orsay, France; d Université Paris-Saclay, Institut d’Optique Graduate School, CNRS, Laboratoire Charles Fabry, Palaiseau, France

**Keywords:** nonmelanoma skin cancer, confocal Raman microspectroscopy, line-field confocal optical coherence tomography, artificial intelligence model, morpho-chemical characterization

## Abstract

**Significance:**

Morpho-chemical characterization of skin cancers provides valuable insights for early diagnosis, classification, and treatment response assessment.

**Aim:**

We introduce a compact, noninvasive system combining high-resolution morphological imaging and chemical characterization of skin tissues. The system integrates line-field confocal optical coherence tomography for cellular-level imaging and confocal Raman microspectroscopy to analyze the chemical composition of specific targets identified within the morphological images.

**Approach:**

We present results obtained from the system installed in a clinical setting over the course of 1 year. More than 330 nonmelanoma skin cancer specimens were imaged *ex vivo*, with different structures targeted for Raman microspectroscopy, resulting in over 1300 spectral acquisitions. To evaluate the system’s ability to accurately identify cancerous structures, an artificial intelligence model was trained on the spectral data.

**Results:**

The model demonstrated high classification performance, achieving an area under the ROC curve of 0.95 for basal cell carcinoma structures and 0.92 when including structures from both basal and squamous cell carcinomas.

**Conclusions:**

Spectral attention scores derived from Raman data revealed key chemical differences among the various cancerous structures, offering deeper insights into their composition.

## 1 Introduction

Skin cancer is the most commonly occurring cancer in humans. It is also the cancer for which the number of cases has increased the most over the last decades. Almost all skin cancers can be divided into two categories: melanoma (10% of skin cancers) and carcinoma or non-melanoma skin cancers (NMSC) (90% of skin cancers). Melanoma arises from melanocytes, which are cells in the skin that produce pigment. NMSC arises from other skin cells called keratinocytes.^1^^,^^2^ NMSC can be divided into two categories: basal cell carcinoma (BCC), corresponding to cancerous basal keratinocytes growing into lobules within the dermis (75% of NMSC), and squamous cell carcinoma (SCC), corresponding to cancerous keratinocytes growing within the epidermis (20% of NMSC). More than 300,000 melanoma are diagnosed each year,^3^ and more than 10 million BCC and 3 million SCC are diagnosed each year,^4^ eventually leading to a higher mortality rate as compared to melanoma,^5^ even though melanoma is more prone to metastasis. In addition to causing negative health outcomes, skin cancer poses a significant economic burden. The factors that most impact prognosis remain early recognition and complete removal of the cancerous tissue before the onset of deep invasion or metastatic disease.^6^

The standard diagnostic procedure is usually based on histological examination and grading of biopsied tissue. To aid early detection of malignant lesions and reduce unnecessary biopsies, noninvasive imaging modalities have been developed, including optical coherence tomography (OCT), reflectance confocal microscopy (RCM), line-field confocal optical coherence tomography (LC-OCT), and multiphoton microscopy (MPM). Conventional OCT provides vertical sections of skin with millimeter-scale fields of view, but it has limited resolution (a few micrometers) and cannot reveal information at the molecular level.^7^ RCM achieves near-histological resolution (∼1  μm). However, its penetration depth is limited to ∼250  μm, and the *en face* image orientation can make interpretation challenging compared with traditional vertically oriented histological sections.^8^ Moreover, RCM lacks the ability to provide molecular information. Combining OCT and RCM allows clinicians to leverage the high resolution of RCM and the deeper tissue penetration of OCT. Clinical studies have shown that integrating these complementary modalities enhances diagnostic accuracy.^9^^,^^10^

LC-OCT is an imaging method based on the principles of both OCT and RCM with line illumination and detection.^11^ LC-OCT has the ability to generate horizontal and vertical section images in real time, as well as volume renderings, with a cellular resolution of ∼1  μm, and a penetration depth of ∼500  μm.^12^^,^^13^ LC-OCT further benefits from a lateral field of view and an imaging depth (1200  μm×500  μm) that are superior to those of RCM but inferior to those of traditional OCT. LC-OCT has shown great potential for improving clinical diagnosis of all types of skin cancers, both NMSC and melanoma.^14^ More recently, LC-OCT has also been shown to have the ability to assist in evaluating preoperative surgical margins for BCC surgery.^15^^,^^16^ However, like RCM and traditional OCT, LC-OCT only provides morphological information about the tissue.

Histology combined with immunohistochemistry, a variant of histology that uses specific protein markers to stain tissues, offers crucial molecular insights for the diagnosis and classification of lesions, particularly for ambiguous lesions or early-stage pathologies with few structural changes. Molecular characterization can also provide information on the tumor environment, aiding in the prediction of the tumor progression and treatment response.^17^^,^^18^

MPM is an imaging modality that leverages nonlinear light–tissue interactions to visualize skin morphology and function at the molecular level. Unlike OCT, RCM, and LC-OCT, MPM provides intrinsic molecular contrast by detecting signals emitted at different wavelengths by various endogenous molecules upon excitation. This capability enables differentiation between tissues based on their molecular composition, offering a unique advantage in characterizing skin structure and pathology.^19^ MPM and RCM have been integrated into a commercially available handheld device. However, the system is limited to vertical cross-sectional imaging and has a relatively small field of view (400  μm×300  μm).^20^ A recently developed MPM-based imaging device enables tissue visualization at a macroscopic scale (ranging from millimeters to centimeters) with subcellular resolution ($0.5\;\mu m^{-1}$). The device can perform second harmonic imaging and time-resolved fluorescence imaging, enabling the detection of endogenous fluorophores such as melanin, elastin fibers, collagen, and keratin.^21^ A key limitation of MPM, however, is its relatively shallow penetration depth compared with interferometric techniques such as OCT and LC-OCT.

Although LC-OCT offers resolution comparable to RCM and MPM, its clinical value could be enhanced by the addition of molecular information to the three-dimensional morphological images. One approach is to couple LC-OCT with confocal Raman microspectroscopy (CRM), a technique that exploits the Raman effect by measuring the spectrum of inelastically scattered light to identify vibrational modes of chemical bonds, thus providing a molecular “fingerprint” of the tissue.^22^^,^^23^ With its confocal setup, CRM enables point measurements in skin tissues, which has proved useful for guiding skin cancer surgery,^24^^,^^25^ as well as for both *ex vivo*^26^^–^^30^ and *in vivo* diagnosis.^31^^–^^33^ A platform for colocalizing LC-OCT images with CRM measurements has been developed previously.^22^ It has been used for characterizing tattoo composition in skin and inflammatory cells of adverse tattoo reaction,^22^ but also on BCC,^34^ for which differences in Raman spectra could be qualitatively observed between BCC lobules, epidermis, and dermis. This platform was limited to *ex vivo* imaging and needed precise calibration subject to drift over time, making its clinical integration complex. To solve this problem, integrating the two modalities in a single device would greatly simplify the co-localization of LC-OCT and CRM. Several devices have already been developed that integrate imaging with Raman spectroscopy,^35^ including OCT with Raman spectroscopy,^36^^,^^37^ OCT with CRM,^38^ full-field OCT with CRM,^39^ RCM with CRM,^40^^,^^41^ RCM, MPM with CRM,^42^ and autofluorescence microscopy with CRM.^43^ Nevertheless, none of these devices provided 3D images compatible with *in vivo* use.

In this paper, we report on a device combining LC-OCT and CRM, compatible with both *in vivo* and *ex vivo* imaging. We present the first large-scale study of Raman spectroscopy guided by cellular-level 3D imaging on *ex vivo* specimens, using a compact probe under conditions compatible with *in vivo* use. The results summarize the imaging and molecular analysis of over 330 skin surgeries: more than 1300 Raman spectra from two types of nonmelanoma skin cancer acquired in 1 year.

As Raman signature illustrates differences in chemical bonds, and as the latter are quite similar across all the molecules contained in biological tissues, only subtle and weak modifications are recorded. These changes in Raman signature need data processing to be identified,^44^^,^^45^ and they do not give direct readable information to the practitioner. Several approaches have been investigated to exploit the chemical information contained in Raman spectra, including using spectra of known components to analyze their contributions to a Raman spectrum and identify its composition,^46^^,^^47^ or classifying Raman spectra by assigning them to pre-identified categories, using metrics of specific Raman peaks identified as relevant chemical bonds,^43^^,^^48^ or using machine learning (ML) or deep learning (DL) techniques.^49^^,^^50^ In the context of skin cancer, DL and ML were used to differentiate Raman spectra of normal versus tumor cells^39^^,^^51^ and to identify the chemical fingerprint for melanoma versus pigmented nevus classification using explanatory modeling.^52^ In clinical settings, DL was applied to data obtained from a portable Raman and autofluorescence system used on *in vivo* patients to perform binary classification of malignant versus benign skin lesions based on convolutional neural networks (CNN),^53^ showing good performance, i.e., a receiver operating characteristic area under the curve (AUC) of 0.96 for differentiation of malignant versus benign tumors as well as melanomas versus pigmented tumors and melanomas versus seborrheic keratosis. Most ML and DL models require large datasets for effective training. To overcome data limitation, DL precisely generative adversarial networks have been used to generate synthetic samples for data augmentation, which has been shown to improve the performance of both DL and ML models.^53^^,^^54^

In the case of skin cancer, it is difficult to apply DL and ML to classify specific, in-depth structures of cancerous lesions if the CRM system is not assisted by 3D image guidance to identify structures in which CRM acquisitions are performed, which is necessary to establish the training dataset. Here, we present a DL model that enables skin cancer structures to be differentiated at the cellular level, directly from freshly excised unprocessed skin tissue. We show that it is possible to accurately classify Raman spectra based on the areas from which they were acquired under LC-OCT image guidance. The DL algorithm achieves an AUC of 0.95 in differentiating for histology-proven BCC lesions between healthy epidermis (EPI-H), epidermis above BCC lobules, healthy dermis (DER-H), and BCC lobules. This high AUC indicates that molecular information can accurately and noninvasively be probed for different targets within skin cancer specimens.

In addition, we propose a method to identify the spectral bands responsible for the distinctions made by the DL model. This approach generates heatmaps that weigh the impact of each wavenumber on differentiation, resulting in attention scores. During biochemical attribution, high attention scores align with the known specific characteristics of each tissue type.

## 2 Materials and Methods

### 2.1 Instrumentation

**Fig. 1:**
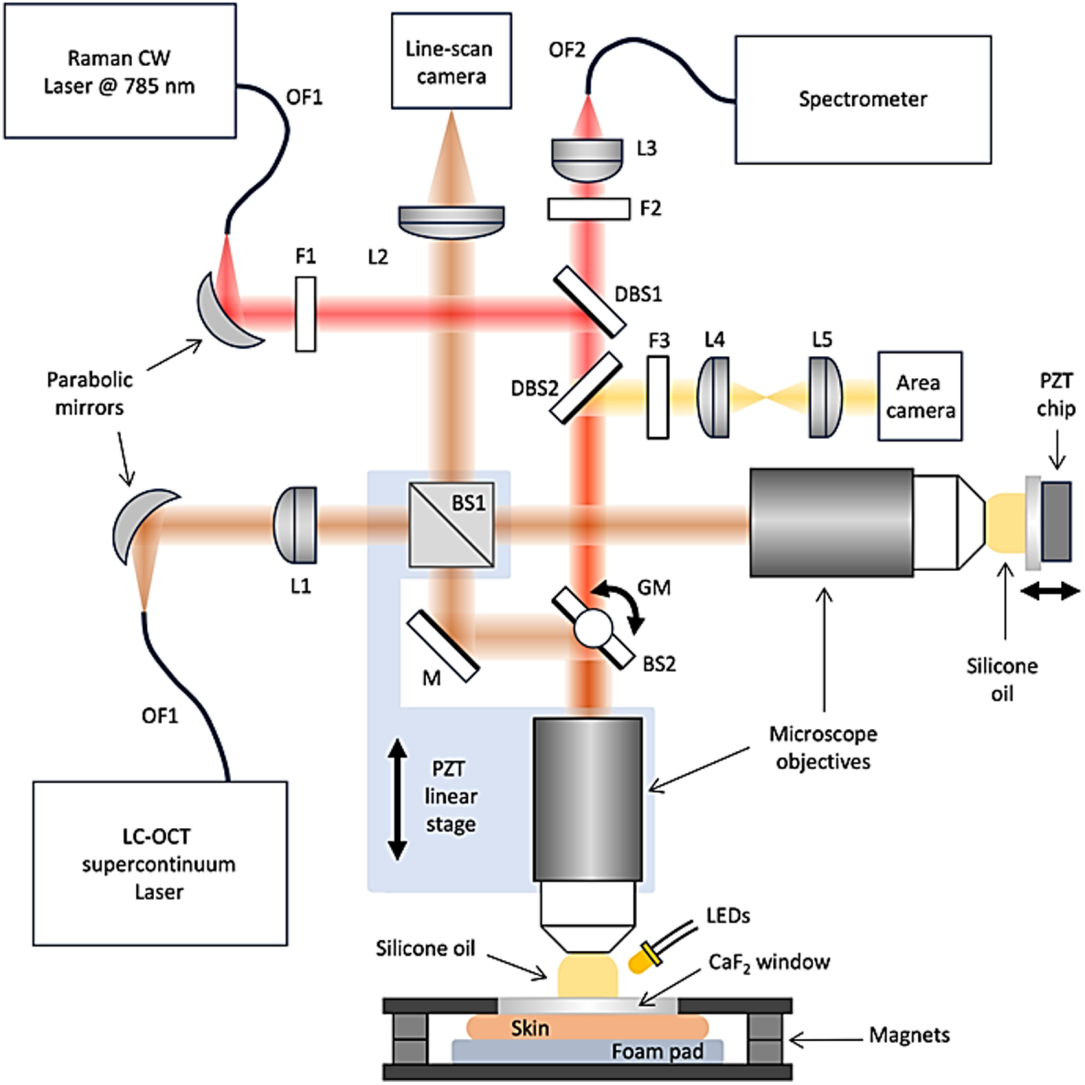
Diagram of the coupled LC-OCT/CRM device and its associated sample holder. BS1: Broadband cube beamsplitter (50R:50T), BS2: Broadband plate beamsplitter (20R:80T), F1: Bandpass filter at 785 nm, F2: Longpass filter at 785 nm, F3: Shortpass filter at 600 nm, DBS1: Longpass dichroïc beamsplitter at 790 nm, DBS2: Longpass dichroïc beamsplitter at 770 nm, OF1: Single mode optical fiber, OF2: Multimode optical fiber, L1: Cylindrical lens, L2, L3: Achromatic doublets, L4 + L5: Afocal optical system, L6: Micro-lens with autofocus, GM: Galvanometer.

The device, integrated into a handheld probe (Fig. 1), comprises three coupled systems: an LC-OCT imaging system, a confocal Raman microspectrometer, and a color surface imaging system. The three systems share the same microscope objective to collect light from the object, thus being intrinsically co-located. LC-OCT and microscopy images are displayed in real time. A Raman spectrum can be acquired at a given point of interest within the field of view of the images.

The LC-OCT imaging system is based on a Linnik-type interferometer with two identical microscope objectives (Olympus, UMPLFLN 20XW, Shinjuku City, Tokyo, Japan). A supercontinuum laser (NKT photonics, Whitelase micro, Birkerød, Hovedstaden, Denmark) is used as a broadband light source at a detected central wavelength of about $\lambda_{0}=800\;\mathrm{nm}$. The beam at the output of a single mode optical fiber (OF1, Thorlabs PM780-HP, Newton, New Jersey, United States) is collimated by a reflective collimator based on a 90 deg off-axis parabolic silver mirror (Thorlabs RCR50A-P01). A cylindrical lens (L1, Edmund 34-648) is employed to generate a line of light focused on the focal planes of the microscope objectives. This line is imaged on the sensor of a line-scan camera (Teledyne e2v, Octoplus, 2048 pixels along the x direction) using a 150-mm focal length achromatic doublet (L2, Thorlabs AC254-150-B). The broadband beam-splitter of the interferometer (BS1, Thorlabs BS017) and the microscope objective in the object arm are mounted on a piezoelectric lead zirconate titanate-driven (PZT) linear stage (Physik Instrument, P625.1CD, Auburn, Massachusetts, United States) for depth (z direction) scanning, whereas a 20% reflectivity mirror (BS2, Edmund Optics 68-370) mounted on a galvanometer (GM, Thorlabs QS7) is placed in the object arm for lateral (y direction) scanning. The focal plane of the microscope objective in the reference arm coincides with the outer side of a 500  μm-thick calcium fluoride (${\mathrm{CaF}}_{2}$) window, thus constituting a low reflectivity (∼3%) reference surface. The window is mounted on a PZT chip (PI PICMA PD080) that oscillates to generate a phase modulation as required to acquire the LC-OCT horizontal section images.^55^ The microscope objectives are immersed in silicone oil whose refractive index (n=1.4) is close to that of skin tissues. The LC-OCT imaging system provides vertical and horizontal section images in real time at a rate of 3 Hz, covering a field of view of 1.0  mm×0.4  mm (x×z) in the vertical imaging mode, and 1.0  mm×0.5  mm (x×y) in the horizontal imaging mode. The acquisition speed is reduced by a factor of ∼3 compared with the conventional LC-OCT setup due to the use of a 20% reflectivity mirror in the LC-OCT path, which limits the amount of light collected from the tissue. With the laser power already set to its maximum, the camera’s integration time is increased to maximize light collection, further limiting acquisition speed. Compared with conventional LC-OCT, the signal-to-noise ratio is only slightly lower and inferior, three-dimensional images can be acquired, with the same quasi-isotropic resolution of ∼1  μm, but over a field of view reduced to 1.0  mm×0.5  mm×0.25  mm (x×y×z).

The Raman microspectrometer is an in-house developed system, using a continuous-wave (CW) laser emitting at the wavelength of 785 nm (Sacher Lasertechnik, Micron Cheetah, Marburg, Germany). The beam at the output of a single-mode optical fiber (OF1, Thorlabs PM780 HP) is collimated using an off-axis parabolic mirror (Thorlabs RCR50A-P01). A sharp band-pass filter at 785 nm (F1, Semrock LL01-785-12.5) removes parasitic wavelengths. The beam is focused on the sample using the same microscope objective as the one employed in the LC-OCT imaging modality. The Raman signal is collected by the same objective. For both excitation and collection, the beam also goes through the beamsplitter BS2, having a transmission of 80%. After collection, the beam is separated from the excitation beam using a long-pass dichroic beamsplitter at 790 nm (DBS1, Chroma RT785RDC). A long-pass edge filter at 785 nm (F2, Semrock LP02-785RU-25) avoids the detection of residual excitation. The Raman signal is focused into a multimode optical fiber (OF2, Thorlabs FG050LGA) using an achromatic doublet (L3, Thorlabs AC127-075-B). The optical fiber both guides the Raman signal to a spectrometer and serves as a confocal aperture. The Raman signal is thus acquired at a point of interest with an estimated resolution of ∼1  μm×1  μm×30  μm (x×y×z). The size of this probed volume results from a compromise between the intensity and spatial resolution of the Raman signal. The spectrometer (Ocean Insight, QE Pro-Raman+, Largo, Florida, United States) has a spectral resolution of $11\;{\mathrm{cm}}^{-1}$. The optical power delivered to the sample is 50 mW. Raman spectra are recorded in a range from 0 to $3000\;{\mathrm{cm}}^{-1}$, with an integration time of 20 s. Raman spectra used in the study consist of the mean spectra of three successive acquisitions. A previous study showed that an acquisition time of 1 min provided a good signal-to-noise ratio (SNR) enabling relevant chemical identification of structures in the skin.^22^ The LC-OCT laser is turned off during Raman acquisition not to generate parasite light collected by the fiber leading to the spectrometer. Each time the sample is changed, and a new series of Raman acquisitions is performed; a first Raman acquisition with the Raman laser switched off is performed to get a spectrum used for background correction of subsequent Raman spectra.

A color surface imaging system is integrated into the system to provide color images of the skin surface. A ring of five white light-emitting diodes (LEDs) is placed around the microscope objective in the object arm of the LC-OCT system to illuminate the skin. Light reflected from the skin surface is collected by the microscope objective, goes through the 80% transmission beamsplitter BS2 as the Raman beam, and is separated from it by a long-pass dichroic mirror reflecting the wavelength below 770 nm. An afocal optical system (L4 + L5) projects the surface image on the area sensor of a color camera equipped with an autofocus micro-lens (IDS Imaging Development Systems, uEye XS, Obersulm, Germany). A short-pass filter at 600 nm (F3, 84-697 Edmund) eliminates most of the stray light from the supercontinuum laser, allowing the surface images and the LC-OCT images to be acquired simultaneously. The surface image has a spatial resolution of 5  μm and covers a field of view of 2.5 mm in diameter. The LEDs are turned off during Raman acquisition.

Custom software displays the images and allows for Raman acquisitions. Raman spectra are acquired at a fixed point (x,y) in a horizontal LC-OCT image. A graphic marker (cross) is displayed on the image to indicate the position of the point where a Raman acquisition can be performed. The depth (z) of Raman acquisition is the same as the depth at which the horizontal LC-OCT image is acquired, controlled by the PZT stage. Similarly, graphic markers are displayed on the surface image to indicate both the region imaged by LC-OCT and the point targeted by the Raman modality. To obtain a Raman spectrum from a given structure, for example, a structure identified within a 3D volume acquisition, the structure must be brought by the user at the position of the point indicated by the graphic marker, and the depth must be adjusted by the user to visualize the structure while imaging in the LC-OCT horizontal mode.

The probe is integrated into a module equipped with a motorized two-axis scanning stage (EK 75×50 Pilot, Märzhäuser Wetzlar), allowing easy positioning of the sample placed on a sample holder attached to the motor, including a manual translation stage (SM1ZM, Thorlabs) for height adjustments (Fig. 1). The top of the sample holder is covered with a 500  μm-thick plate of Raman-grade ${\mathrm{CaF}}_{2}$ (Crystran Ltd, Poole, United Kingdom). Raman-grade ${\mathrm{CaF}}_{2}$ was chosen because its Raman signature consists of a single Raman peak at $321\;{\mathrm{cm}}^{-1}$, minimizing the background Raman signal superimposed on the Raman spectra of skin.^22^ The sample is positioned under the ${\mathrm{CaF}}_{2}$ window using paraffin oil for optical contact and pressed onto it by means of a second plate held to the first one using magnets. Biopsy foam pads are positioned under the sample to avoid altering it. Silicone oil is then positioned between the ${\mathrm{CaF}}_{2}$ plate and the microscope objective. Note that the probe can be easily modified for *in vivo* acquisition by simply adding a tank filled with silicone oil with a ${\mathrm{CaF}}_{2}$ window in contact with the skin.

### 2.2 Protocol

Measurements were carried out *ex vivo* on fresh surgical specimens taken from patients at the dermatology department of Saint-Etienne University Hospital, France, between May 2023 and August 2024. The study was conducted with the informed consent of patients and with the approval of the Research Ethics Committee (IRBN522023/CHUSTE).

Each surgical specimen was analyzed immediately after excision and after being cleaned of blood using physiological fluid. The specimen was placed in the sample holder positioned under the LC-OCT Raman probe (Fig. 1). Optical contact between the ${\mathrm{CaF}}_{2}$ window and the skin sample was achieved using a very thin layer of paraffin oil. The sample was oriented with its surface facing the ${\mathrm{CaF}}_{2}$ window. A plate was added under the sample, and its position was adjusted using a retaining magnet to gently press the sample against the upper window, thereby preventing any sample displacement during experiments. Silicone oil was added to the top of the sample holder as an immersion medium. Silicone oil has a refractive index of 1.4, close to the refractive index of skin, which ensures that the coherence plane and the focal plane of the objective are superimposed during depth scanning, thus maintaining optimum resolution and signal at all depths. Regarding CRM acquisition, silicone oil is far enough from the focal volume of the microscope objective not to parasite the Raman signal, and the layer of paraffin oil on the sample is so thin that it does not affect either the Raman signal. For every specimen, before applying the imaging protocol, a CRM acquisition was performed with the CRM laser turned off for background subtraction in Raman spectra.

For each specimen, a 3D LC-OCT image was acquired in a region presenting tumor criteria. Raman spectra were then acquired at several points of interest (POI) in this region. The targeted POI depends on the type of lesion. For BCC, the targets are the epidermis above the tumoral epidermis (EPI-T) and the BCC tumor lobules (LOB-T). For SCC, the target is the tumor (TUM-T), i.e., the cancerous keratinocytes within the epidermis. Raman spectra were also acquired remotely in healthy regions of the epidermis (EPI-H) and dermis (DER-H). The healthy part corresponds to the margins of the surgical specimen. Six spectra are acquired for each surgical specimen. The specimens were then sent for histopathological analysis for confirmation of the cancer type. For histopathologically confirmed lesions, *a posteriori* verification of the POI probed with CRM within the 3D LC-OCT images was performed by an experienced dermatologist to confirm that the Raman spectra come from POI fully included in the associated target.

**Fig. 2:**
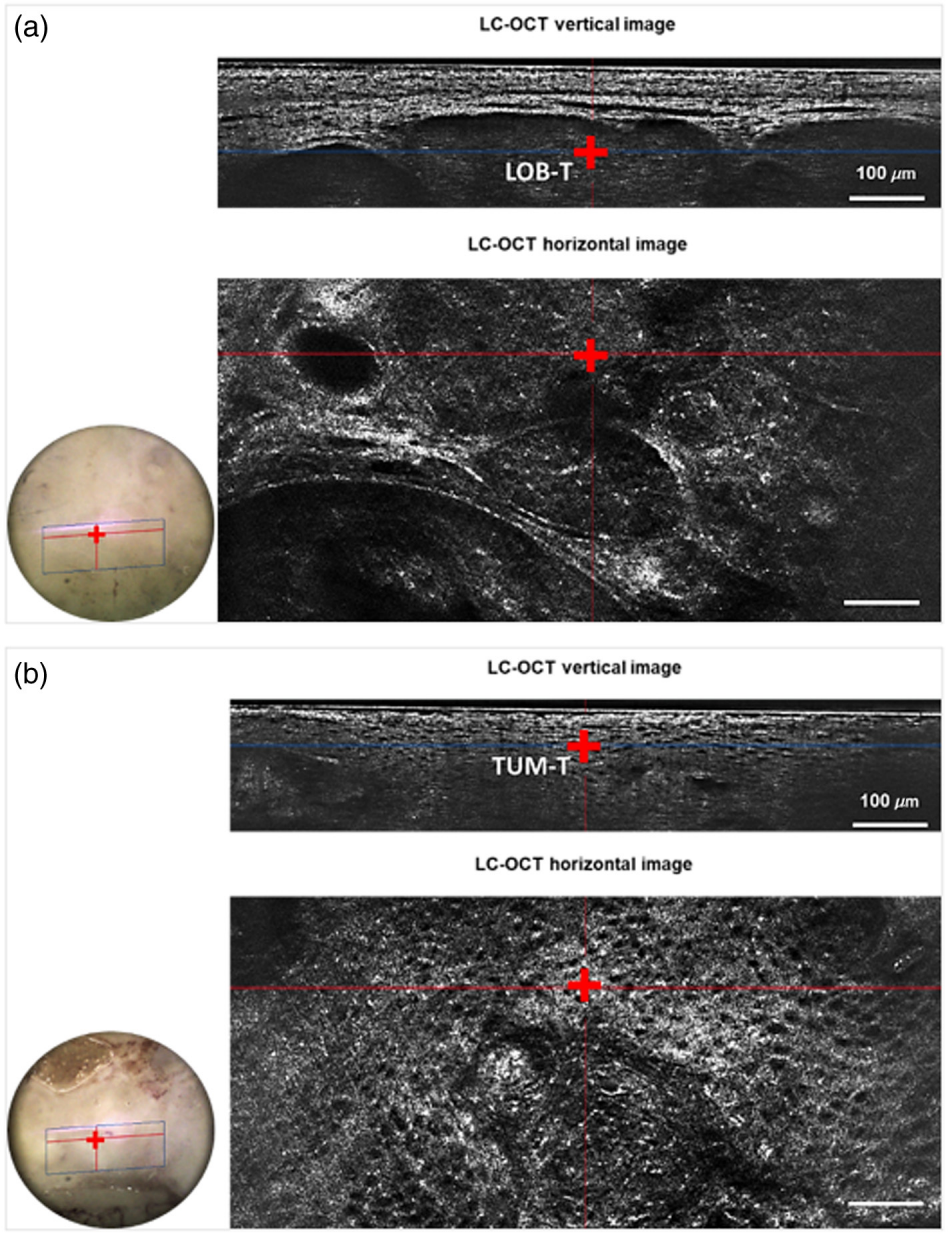
Example of 3D LC-OCT images of different CRM targets: (a) for basal cell carcinoma (BCC) at the BCC tumor lobule (LOB-T), and (b) for squamous cell carcinoma (SCC) at the tumor (TUM-T). The point probed by CRM is represented by the red cross on the images. The circular images to the left of the 3D LC-OCT images represent the surface image of the surgical specimens.

The data have been acquired from lesions of 332 patients, including 213 males and 119 females with a median age of 77 years old (range: 30 to 96) and 81 years old (range: 44 to 99), respectively. Of the 332 lesions, 247 cases were BCC and 85 SCCs. All these lesions were clinically confirmed by the experienced dermatologists and by histopathology. On 247 BCC surgical specimens, a total of 1069 Raman spectra were selected: 364 LOB-T, 253 EPI-T spectra, 236 EPI-H spectra in the *in sano* zone and 216 DER-H spectra in the *in sano* zone. On 85 SCC surgical specimens, a total of 313 Raman spectra were selected: 219 TUM-T spectra and 94 EPI-H spectra in the *in sano* zone. Table 1 summarizes the number of specimens and spectra analyzed for each lesion type and target. Figure 2 presents examples of 3D LC-OCT images with targets for CRM acquisition, for the two types of lesions analyzed, i.e., BCC and SCC.

**Table 1:** Number of specimens and spectra analyzed for each lesion type and target.

Lesion	Number of lesions	Target	Number of spectra
BCC	247	EPI-T	253
		LOB-T	364
		EPI-H	236
		DER-H	216
SCC	85	TUM-T	219
		EPI-H	94
Total lesions	332	Total spectra	1382

### 2.3 Raman Spectra Processing

To extract the most relevant Raman features from the raw data and minimize the impact of measurement artifacts, a preprocessing procedure was optimized. After CRM acquisition, all spectra were divided into three spectral ranges (800 to 1350, 1350 to 1750, and 2800 to $3000\;{\mathrm{cm}}^{-1}$), and the following preprocessing steps were applied:

1. extension of the range by $50\;{\mathrm{cm}}^{-1}$ at both ends to avoid edge effects
2. baseline correction using polynomial fitting of order 5 for the first two ranges and 1 for the last range with asymmetric quadratic function with tolerance 0.01 as cost function^56^
3. removal of the extended margins added in step 1
4. standard normal variate (SNV) normalization
5. application of Savitzky–Golay filter (second order, window size 13 points).

After applying this preprocessing method to the three ranges separately, the data were concatenated to form the final input data to the models described below. The average spectra for all of the different targets are shown in Fig. S1 in the Supplementary Material. Shaded areas indicate the standard deviation of all spectra. The average spectrum of all targets combined is also shown on each graph, to reveal deviations in the spectrum of a given target and highlight its particularities.

### 2.4 Artificial Intelligence Models and Spectral Attention Score

Artificial intelligence (AI) models have been developed to classify the different targets analyzed by CRM. The performance of standard ML methods was compared with a simple 1d-CNN. The ML models evaluated were: XGBoost^57^ with 100 estimators and a maximum depth of 5; k-Nearest Neighbors (k-NN) with 10 neighbors and Euclidean distance; multilayer perceptron (MLP) with hidden layers of sizes 32 and 16; and support vector machine (SVM) with a radial basis function (RBF) kernel. The CNN consists of three convolution layers (kernel sizes: 7, 3, and 3; strides: 2; features: 16, 32, and 64), each followed by group normalization^58^ with four groups and rectified linear unit (ReLU) activation. The features are then flattened and passed through three fully connected layers (output features: 256, 64, and 5) with batch normalization^59^ and ReLU activation.

Following standard practice, a fivefold cross-validation scheme was employed. The folds were stratified by labels, with patients grouped to ensure no individual appeared in both training and validation sets. Two types of multiclass models were developed, with four classes for BCC targets and five for BCC and SCC targets, as described in Sec. 2.2.

To identify key features in the input spectra influencing class attribution, we used the AI model to generate spectral attention scores from Raman spectra. These scores highlight spectral regions relevant for classifying targets. Raman band shifts or intensity changes, linked to protein and lipid content, can indicate metabolic changes and potential skin cancer biomarkers.^60^ We applied an occlusion method to refine this process, replacing input regions with zeros and measuring output variation.^61^ Regions causing the greatest variation were deemed most important. This was performed using a sliding window of size 13 and stride 5. The absolute variations were normalized to a 0 to 1 scale using min–max normalization.

Note that this was computed for all out-of-fold samples for all folds and then averaged to yield robust estimates of the attention scores. To quantitatively assess consistency across folds, we computed the pair-wise cosine similarity between the average attention score vectors for all pairs of folds, for each target class. We then averaged these values to obtain a similarity score for each class. The values that we obtained are shown in Table S1 in the Supplementary Material. These high values testify to the consistency of the attention scores considered.

**Fig. 3:**
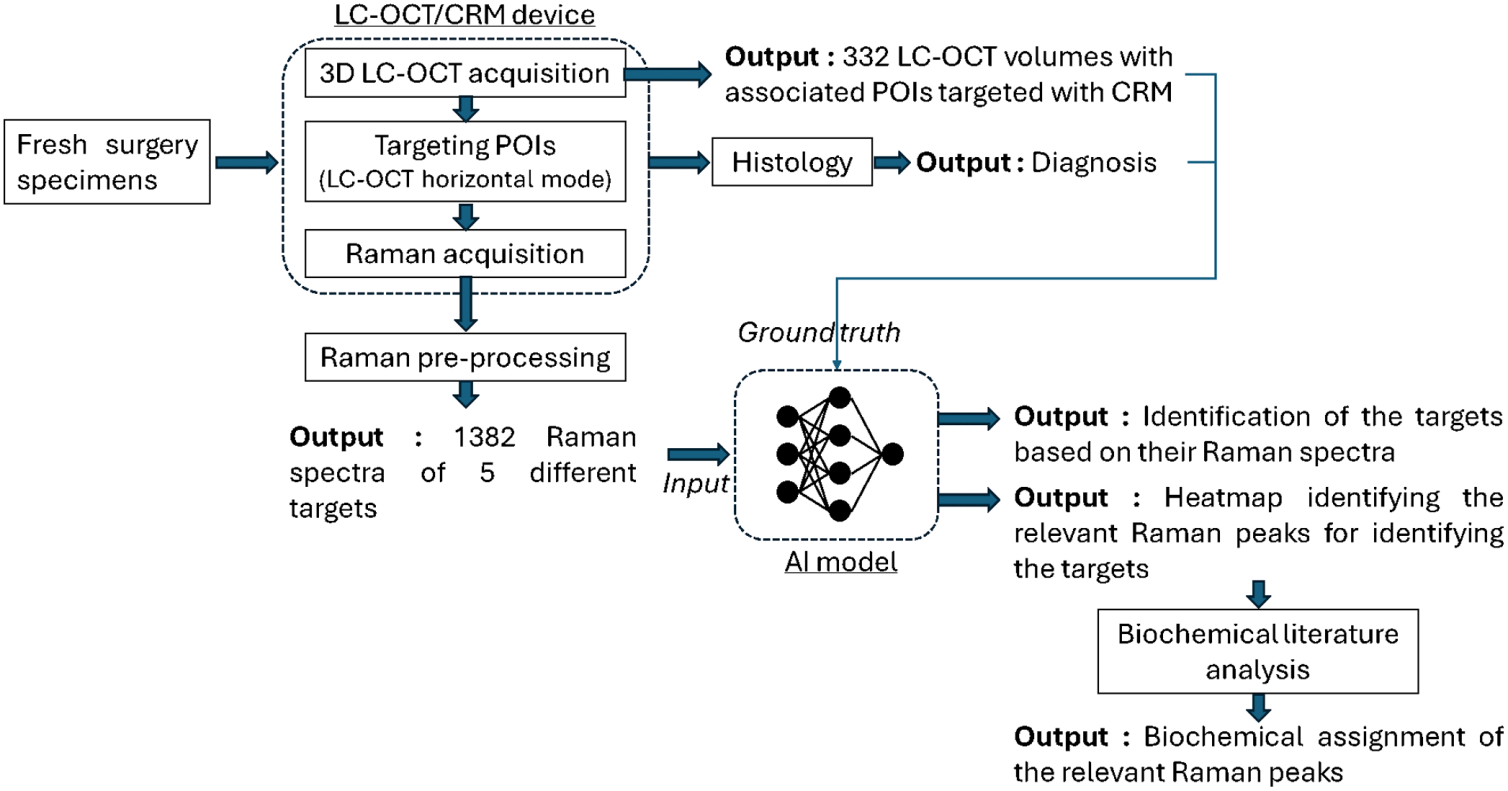
Visual flowchart summarizing the protocol and its outputs.

The flowchart in Fig. 3 summarizes the experimental workflow from data acquisition to interpretation of results.

## 3 Results

### 3.1 AI Performance

We show in Table S2 in the Supplementary Material the performance (AUC) of the different AI methods described in Sec. 2. AUC values for multiclass models correspond to the average of the class-wise receiver operating characteristic AUC values from binary “one-vs-rest” classification tasks. The best-performing method, the 1d-CNN model, was selected for further analysis.

For more details about 1d-CNN models performance, Fig. S3 in the Supplementary Material presents confusion matrices for each one-versus-rest task, and Table S3 in the Supplementary Material shows key performance metrics (AUC, recall, sensitivity, specificity, accuracy, and precision) computed with 1d-CNN models. Confusion matrices for multiclass setups are built using the maximum score’s class and built using a threshold of 0.5 for binary setups.

**Fig. 4:**
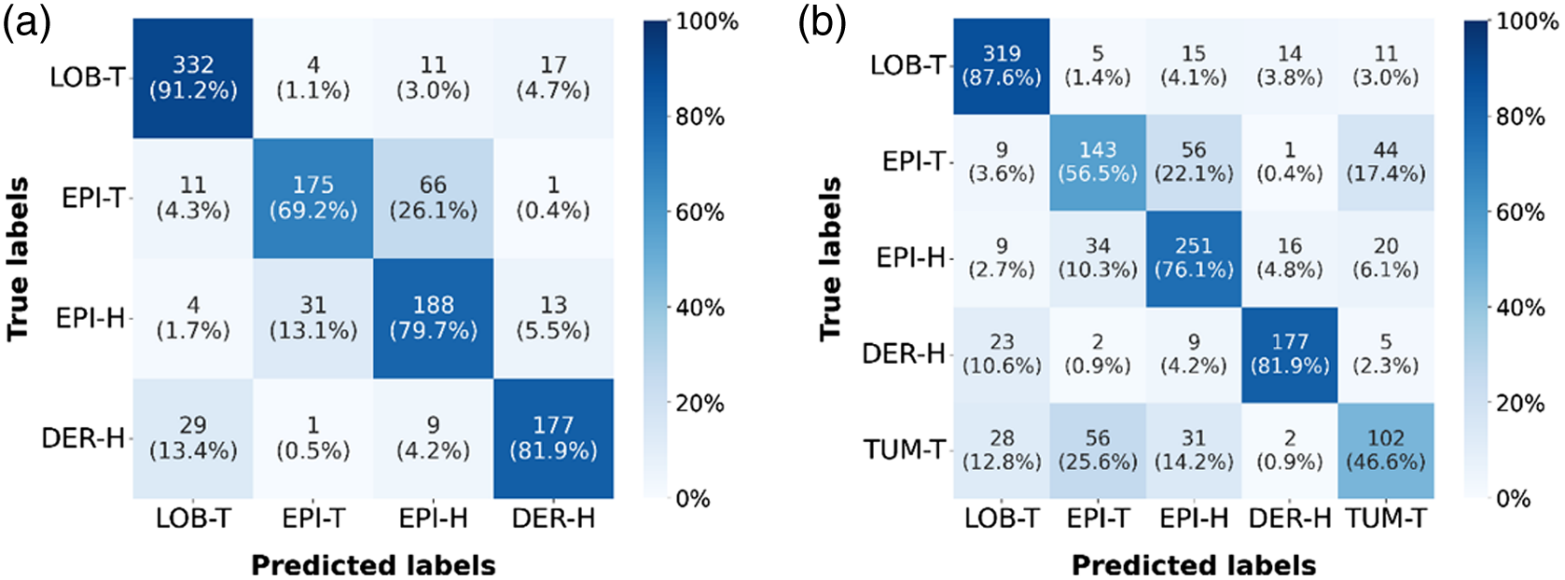
Confusion matrix associated with the 1d-CNN models developed for (a) BCC targets and (b) BCC and SCC targets. Normalization is performed row-wise. Each cell shows the raw count along with the proportion of samples predicted as that class among all samples with the given ground truth class (in parentheses).

For BCC targets, we obtain an AUC of 0.95. The associated confusion matrix is given in Fig. 4(a). The confusion matrix shows the performance of the model, indicating for a given target what the model predicted, both in absolute value (number of spectra) and as a proportion of all spectra for the given target. For example, 91.2% of the LOB-T spectra (332 spectra) were correctly predicted as LOB-T, whereas 8.8% (32 spectra) were incorrectly labeled, 4 being labeled as EPI-T, 11 as EPI-H, and 17 as DER-H. Note that the EPI-H and DER-H are limited to acquisitions for BCC lesions. The model best performs for discrimination of BCC lobules and DER-H. Only 13.4% (29) of DER-H samples were misclassified as LOB-T. This confusion can be due to close spectral signatures, linked to similar biochemical components (lipids, proteins such as collagen: amide I at $∼1650\;{\mathrm{cm}}^{-1}$, proline/hydroxyproline at ∼850 to $950\;{\mathrm{cm}}^{-1}$) or spatial overlap between dermis and LOB-T.^62^^,^^63^ This confusion can also be explained by the presence of identical deoxyribonucleic acid/ribonucleic acid (DNA/RNA) despite different organizations or by partial overlap of the analyzed areas. Regarding the epidermis, 26.1% of the spectra of the epidermis in the tumor area were predicted as EPI-H, and conversely, 13.1% of the spectra of EPI-H were predicted as epidermis in the tumor area. Nevertheless, 69.2% (resp. 79.7%) of spectra of epidermis in the tumor area (resp. EPI-H) were correctly identified by the model. It is important to note that EPI-H exhibits the same morphological features as the epidermis in the tumor area, showing an interest in chemical characterization using CRM.

For BCC and SCC targets, we obtain an AUC of 0.92. The associated confusion matrix is shown in Fig. 4(b). The introduction of the SCC targets (including TUM-T and EPI-H) marginally affected the performance of the model in identifying BCC lobules (87.6% of correct predictions) and DER-H (81.9% of correct predictions) compared with the model developed for BCC alone. For this model, EPI-H targets from SCC were merged with EPI-H targets from BCC in a single EPI-H class. It is interesting to note that the performance for identification of EPI-H was slightly lower in this model (76.1% versus 79.7% in the model developed for BCC), whereas it was degraded for the epidermis in the BCC area, which becomes more prone to confusion with cancerous SCC epidermis (17.4% of the EPI-T spectra become predicted as TUM-T). Conversely, TUM-T is prone to confusion with EPI-T (25.6% of TUM-T spectra are predicted as EPI-T). TUM-T and particularly EPI-T are prone to confusion with EPI-H in this model. As observed for BCC, distinguishing between the different types of epidermis (healthy, SCC, and in the area of BCC) is confirmed as not being straightforward, despite the decent performance of the model. Of the SCC tumor (TUM-T) samples, 14.2% (31) were misclassified as EPI-H.^64^ Well-differentiated SCCs produce keratins and proteins similar to EPI-H (amides I/III ∼1650, $∼1240\;{\mathrm{cm}}^{-1}$),^65^ making their Raman signatures virtually identical and complicating their distinction. This biochemical and spectral similarity between healthy and tumoral keratinocytes explains the difficulties of diagnosis by Raman spectroscopy, particularly for low-grade SCCs, which retain an architecture close to that of healthy tissue.^64^

### 3.2 Biochemical Analysis

**Fig. 5:**
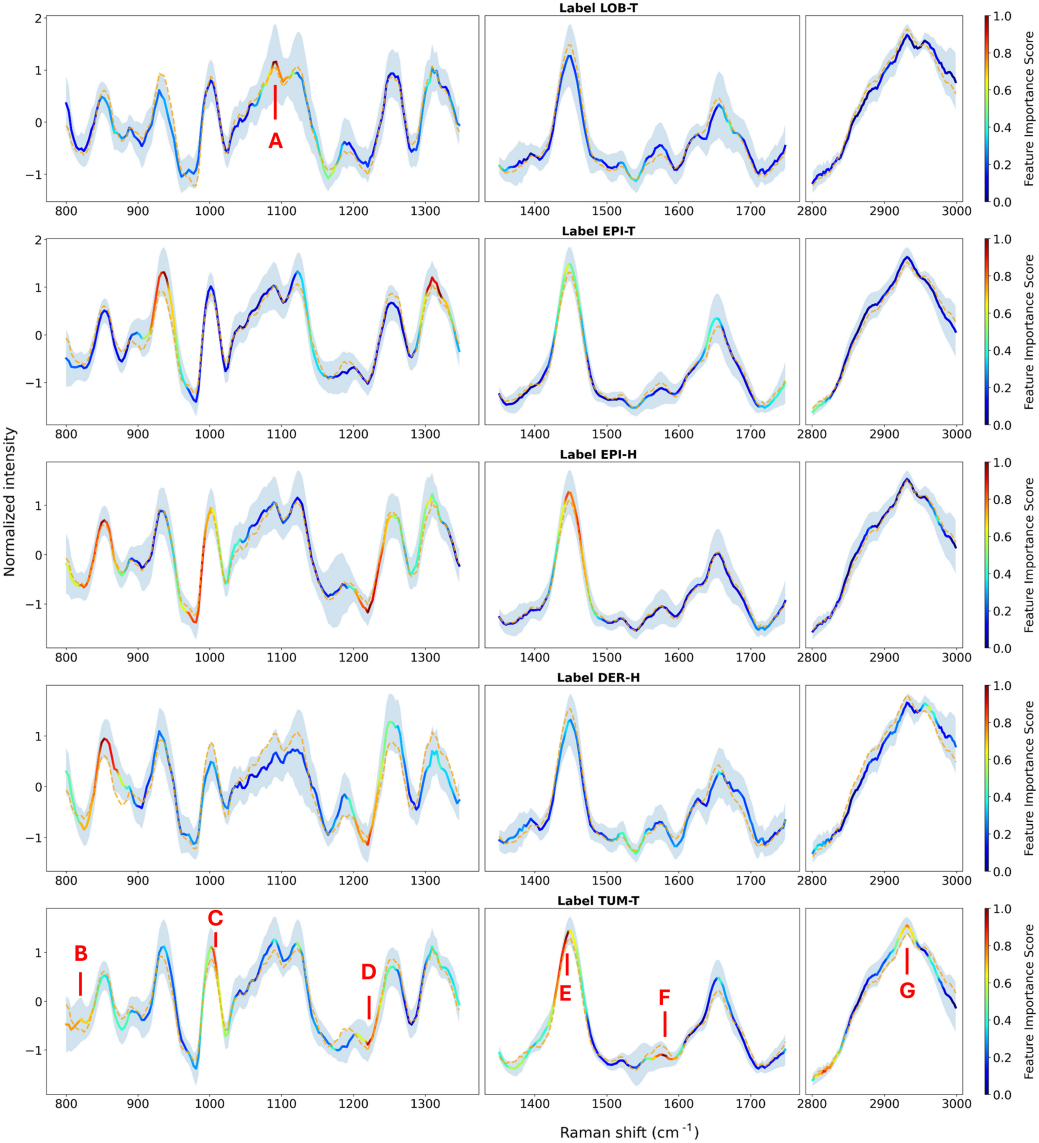
Heatmaps of attention scores for Raman spectra of: the BCC tumor lobules (label LOB-T), the epidermis above BCC tumor (label EPI-T), the healthy epidermis (label EPI-H), the healthy dermis (label DER-H), and the tumor of the SCC (label TUM-T). Annotations A to G (in red) highlight areas of interest. The orange dashed curve represents the mean spectrum of all the targets combined.

The chemical assignments of the Raman bands can indicate the presence of specific biomolecules that serve as potential biomarkers for skin cancer. Shifts in band positions or changes in intensities can be associated with protein and lipid content, reflecting underlying metabolic changes.^60^ By analyzing the average Raman spectra from the different lesion types and targets, combined with the spectral attention scores generated from the AI model, distinct spectral regions and chemical compositions that differentiate healthy from cancerous tissues were identified. Figure 5 also displays the average spectrum of all the targets combined (orange dashed curve), to locate deviations in the spectrum of a given target, pointing out the particularities of that spectrum. Shaded areas indicate the standard deviation of all spectra to show the measured consistency across multiple patients and measurements. The assignment of bands was conducted based on an extensive literature review. The vibrational groups and modes were associated with specific skin components, as detailed in Table 2. An exhaustive listing of the correspondence between all the group and vibrational modes and their associated biochemical assignments in the skin, obtained from the literature review, is included in Table S4 in the Supplementary Material.

**Table 2:** Biochemical assignments of the average spectrum based on literature. ν, stretching; $\nu_{s}$, symmetric stretching; $\nu_{\mathrm{as}}$, antisymmetric stretching; δ, bending; τ, twisting.

Raman shift (${\mathrm{cm}}^{-1}$)	Discriminations	Group and vibrational mode	Biochemical assignments	References
821	SCC epidermis (B)	ν(C–C)	Proline and hydroxyproline (collagen)	66, 67
1001	SCC epidermis (C)	$\nu_{s}(C-C)$ Ring breathing	Phenylalanine (collagen)	66, 68–70
1092 to 1109	BCC lobules (A)	$\nu_{s}$(O–P–O)	DNA backbones (nucleus)	47, 71
1450	SCC epidermis (E)	$\delta_{\text{scissoring}}$ (CH)	Proteins (collagen), lipids	48, 49, 72–75
1573	SCC epidermis (F)	δ(N–H), ν(C–N)	Nucleic acids DNA	25, 66, 76, 77
2930	SCC epidermis (G)	$\nu_{s}$(${\mathrm{CH}}_{3}$)	Mostly keratin	78, 79

Regarding BCC lobules (LOB-T), the upper part of the band centered around $1092\;{\mathrm{cm}}^{-1}$ (annotated A Fig. 5), corresponding to the symmetric stretching of ${\mathrm{PO}}_{2}$ from DNA, appears essential for classification. The 1025 to $1165\;{\mathrm{cm}}^{-1}$ region, a spectral region of interest for DNA,^47^^,^^71^ was extracted from the average spectra of each target, deconvoluted and integrated, as shown in Figs. S2(A)–S2(F) in the Supplementary Material. Deconvolution was performed using Orange Data Mining software version 3.34.0, with the Spectroscopy 0.6.9 Add-on. Gaussian fitting components were applied for deconvolution to minimize the reduced chi-square value.

Notably, a component centered at $1089\;{\mathrm{cm}}^{-1}$ showed significant variability, with the integration value (${\mathrm{AUC}}_{1089}$) 70% higher than that of EPI-H. This finding aligns with the increased DNA content associated with the high proliferative activity observed in BCC cells.^68^^,^^80^ In addition, the BCC lobules category exhibited a small component at $1092\;{\mathrm{cm}}^{-1}$ that was absent in the other categories; however, the corresponding vibrational group was not identified.

Regarding SCC tumor (the TUM-T category), six discriminant regions were identified centered at 821, 1012, 1220, 1446, 1580, and $2931\;{\mathrm{cm}}^{-1}$, labeled from B to G (Fig. 5), respectively. Most of these regions (Fig. 5 B, C, E, and G) are primarily linked to proteins, particularly collagen and keratin. These peaks exhibit higher-than-average intensity, suggesting an overexpression of proteins. The high wavenumber region around $2931\;{\mathrm{cm}}^{-1}$ (Fig. 5 G) was further deconvoluted into five separate components. The specific component corresponding to the highest attention score is related to the symmetric stretching of ${\mathrm{CH}}_{3}$ groups, which is linked to keratin.^78^ The integration value ${\mathrm{AUC}}_{2931}$ for SCC epidermis was 44% higher than that of EPI-H, aligning with previous findings that indicate SCC contains higher levels of keratin, ceramides, and water compared with healthy skin.^68^^,^^80^ This keratin attribution is further supported by the lower ${\mathrm{AUC}}_{2931}$ values observed in BCC lobules and DER-H, both of which are less dense in keratinocytes.^79^

Lipid content is a key indicator of differentiation, varying according to the specific skin layer and overall skin homeostasis. Lipid content was estimated by comparing the sum of areas of two lipid-attributed components centered at $2855\;{\mathrm{cm}}^{-1}$ (width: $30\;{\mathrm{cm}}^{-1}$) and $2880\;{\mathrm{cm}}^{-1}$ (width: $20\;{\mathrm{cm}}^{-1}$) (${\mathrm{AUC}}_{2840-2870}$ and ${\mathrm{AUC}}_{2870-2890}$) to those attributed to keratin, centered at 2930 and $2955\;{\mathrm{cm}}^{-1}$ (both with a width of $20\;{\mathrm{cm}}^{-1}$) (${\mathrm{AUC}}_{2920-2940}$ and ${\mathrm{AUC}}_{2945-2965}$) according to the formula: $\text{Lipid content}=\frac{{\mathrm{AUC}}_{2840-2870}+{\mathrm{AUC}}_{2870-2890}}{{\mathrm{AUC}}_{2920-2940}+{\mathrm{AUC}}_{2945-2965}}$, as described by Li et al.^69^

The results, illustrated in Fig. S2(G) in the Supplementary Material, indicate higher lipid concentrations in the epidermis (healthy, SCC, or BCC) and lower concentrations in BCC lobules and DER-H. These estimations are also consistent with the expected lipid distribution across the different skin layers.

The spectral attention scores generated based on the AI model highlighted specific spectral areas that were closely aligned with the compositional differences among tissue categories, indicating a nonrandom and targeted correlation.

## 4 Discussion and Conclusion

We have presented an innovative device combining LC-OCT and CRM to characterize nonmelanoma skin cancers. The device, integrating three channels to provide LC-OCT imaging, CRM, and color surface imaging, is compatible with both *in vivo* and *ex vivo* imaging while retaining the overall performance of the LC-OCT and CRM modalities. A specific *ex vivo* module with a sample holder adapted for both LC-OCT and CRM has been introduced. In this first large-scale *ex vivo* study of 3D image-guided Raman spectroscopy at the microscopic scale, we used a compact probe on fresh, unprocessed *ex vivo* skin tissues. Although the device is compatible with *in vivo* imaging, the current relatively long acquisition time for Raman spectra (1 min) makes it difficult to carry out *in vivo* studies. A future study could compare the performance of the AI model with reduced acquisition times, collecting more BCC spectra.

Measurements were carried out on fresh surgical specimens of different types of skin cancer taken from 332 patients at the dermatology department of Saint-Etienne University Hospital, France. The processing and analysis of a large number of Raman spectra (1382 Raman spectra acquired over 1 year) required the development of processing tools capable of retaining the most relevant information from the raw data. For this reason, we have introduced a DL algorithm to differentiate skin cancer structures at the cellular level. Our results show that it is possible to accurately classify Raman spectra according to the areas from which they were acquired under LC-OCT image guidance. We also used a method to identify the spectral bands responsible for the distinctions made by the AI model. This approach can generate heat maps showing the impact of each wavenumber on differentiation based on attention scores. During biochemical assignment, high attention scores align with the known specific characteristics of each tissue type.

To validate our model, an initial analysis was carried out on the Raman spectra of the BCC targets (EPI-T, LOB-T, EPI-H, and DER-H). These targets are well distinguished thanks to the morphological structure of the BCC, which is well defined when imaged using LC-OCT.^81^ For BCC, the DL model achieved an AUC of 0.95 to differentiate between EPI-H, epidermis above BCC lobules, and DER-H and BCC lobules. This high AUC indicates that molecular information can be probed accurately and noninvasively for different targets in skin cancer samples. This analysis showed that it is possible to discriminate correctly between BCC lobules and DER-H, whereas it was less obvious to discriminate between EPI-T and EPI-H. Of epidermal spectra in the tumor zone, 24.9% were predicted as EPI-H, and conversely, 14.8% of healthy epidermal spectra were predicted as epidermis in the tumor zone. This could be attributed to a molecular content more similar between the EPI-H and the tumor epidermis than between the lobules or the tumor dermis. This could also be linked to the fact that EPI-H is sometimes part of the cancerization process and therefore difficult to discriminate from epidermis at the tumor level in those cases. Nevertheless, 69.2% (resp. 79.7%) of the spectra of the epidermis in the tumor zone (resp. EPI-H) were correctly identified by the model, whereas it should be noted that the epidermis has the same LC-OCT morphological appearance in those two cases. Given the labeling uncertainty—which is difficult to fully eliminate—there is a potential for model bias, particularly a reduced sensitivity in tumor detection if areas undergoing early cancerization are labeled as healthy. To mitigate this, methods such as label smoothing or pseudo-labeling on identified noisy-labeled samples could be explored.

The chemical assignment of Raman bands indicating the presence of specific biomolecules that serve as potential biomarkers of skin cancer shows that for BCC lobules, the upper part of the band centered around $1092\;{\mathrm{cm}}^{-1}$ corresponding to the symmetrical stretching of ${\mathrm{PO}}_{2}$ DNA, appeared essential for classification. The component centered at $1089\;{\mathrm{cm}}^{-1}$ showed significant variability, with an integration value (${\mathrm{AUC}}_{1089}$) 70% higher than that of EPI-H. This result was consistent with the increased DNA content associated with the high proliferative activity observed in BCC cells.^68^^,^^80^

BCC served as a relevant proof-of-concept to show that the introduced system yields accurate chemical characterization based on Raman spectra when used in conjunction with a DL model. Nevertheless, LC-OCT alone has been shown to perform very well for accurate diagnosis and subtyping of BCC,^82^ whereas Raman characterization would be more relevant to provide an additional layer of chemical subtyping, which could for example be relevant for personalized treatment options to be explored in further studies.

When including SCC targets in addition to BCC targets, the model gave an AUC of 0.92. As in the case of BCC, the distinction between the different types of epidermis (healthy, SCC, and in the area of the BCC) was confirmed as difficult, which may be explained by the fact that the chemical composition of the epidermis does not change significantly between the different targets or that the changes are too subtle to be efficiently classified based on CRM. As for BCC, areas we considered healthy could also in fact be part of the cancerization process, leading to inaccurate labeling for the DL model. From a biomolecular perspective, six discriminant regions were identified for the SCC epidermis centered at 821, 1012, 1220, 1431, 1585, and $2931\;{\mathrm{cm}}^{-1}$. Most of these regions are mainly protein-related, especially collagen and keratin. These peaks are above average, suggesting overexpression of proteins. The region of the large number of waves around $2931\;{\mathrm{cm}}^{-1}$ corresponding to the highest attention score is related to the symmetrical stretching of ${\mathrm{CH}}_{3}$ groups, which is related to keratin. The ${\mathrm{AUC}}_{2931}$ integration value of SCC epidermis was 44% higher than that of EPI-H, which is consistent with previous results indicating that SCC contains higher levels of keratin, ceramides, and water compared with healthy skin.^68^^,^^80^ This allocation of keratin is further supported by the lower ${\mathrm{AUC}}_{2931}$ values observed in BCC lobules and DER-H, both of which are less dense in keratinocytes.^79^

Even though our study comprises a large number of lesions, the number of spectra is limited by the need for annotation. Alternative approaches, such as semi-supervised learning—which combines carefully annotated spectra with large amounts of unlabeled spectra—could be explored in future work. For instance, Rui et al.^83^ showed that leveraging unlabeled data can lead to improved accuracy compared with a fully supervised approach.

In conclusion, this new device combining LC-OCT and CRM, compatible with *in vivo* and *ex vivo* imaging, assisted by a dedicated AI model, proved capable of discriminating BCC structures, even for structures with similar morphological features. Encouraging results were obtained for SCC, although the differentiation of epidermal structures currently lacked accuracy. Considering the capability of the device for chemical differentiation of well-defined structures, in particular for BCC, future investigations using the developed system could in particular include chemical subtyping of BCC to obtain a new layer of information that could guide therapy options, or assist in developing them. Using the chemical information provided by CRM could also be useful for identifying the nature of unusual structures that could be considered BCC lobules on LC-OCT imaging examination. Finally, future technical developments should facilitate the use of the system *in vivo*, in particular by reducing the acquisition time of Raman spectra.

## Supplementary Material

10.1117/1.JBO.30.7.076008.s01

## Data Availability

The authors affirm that all data necessary for confirming the conclusions of the article are present within the article, figures, and tables.
