# Supplementary material for: AI-assisted identification of nonmelanoma skin cancer structures based on combined line-field confocal optical coherence tomography and confocal Raman microspectroscopy
Source: J Biomed Opt. 2025 Jul 28;30(7):076008. doi: 10.1117/1.JBO.30.7.076008 (PMC12302994; doi:10.1117/1.JBO.30.7.076008)
Supplement: Supplementary file 1 [file JBO_030_076008_SD001.docx]

**Supporting Information**


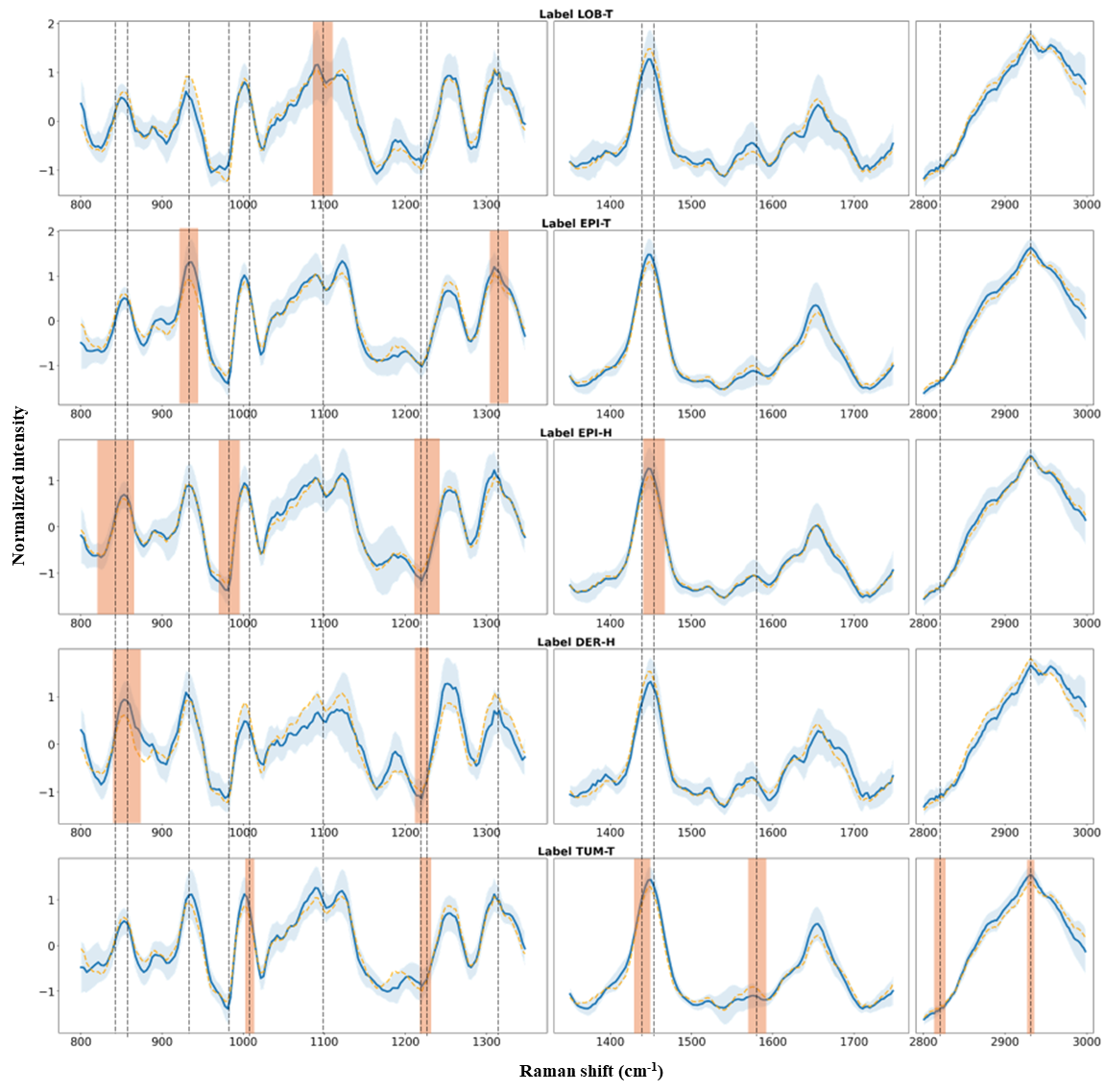


Figure S1: Mean spectra for the different targets. The blue curve represents the average spectrum across all categories, along with its standard deviation. The yellow dashed curve represents the mean spectrum of all the targets combined. Orange boxes represent the parts of the spectra identified as relevant for differentiation by the developed AI model. Dashed lines show the center position of the boxes across all the spectra.

**
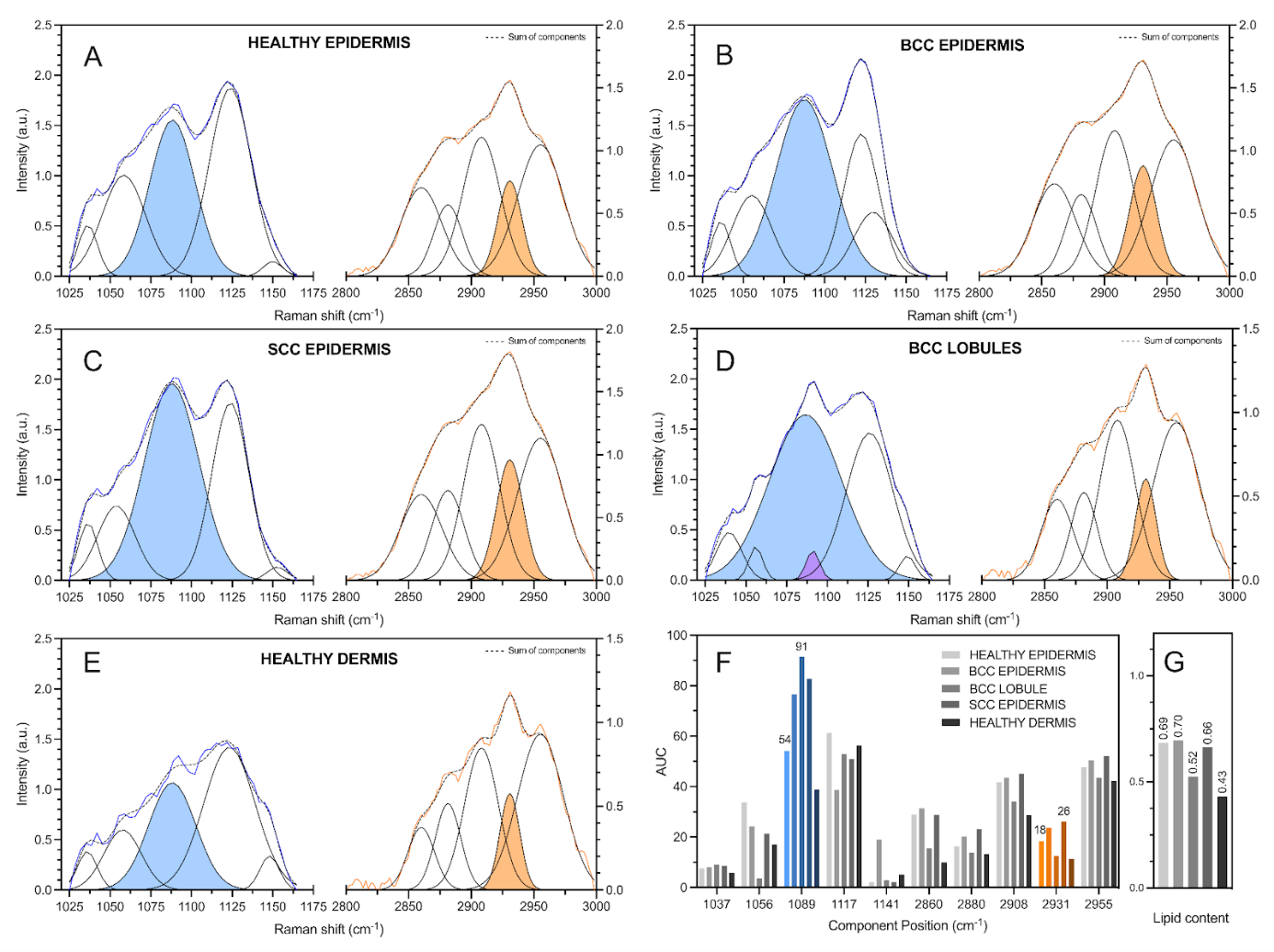
**

*Figure S2: Deconvolution components of the regions 1025 – 1165 cm^-1^ (left) and 2800 – 3000 cm^-1^ (right). Solid colored curves represent the average spectral data from each category, while dashed curves indicate the sums of all presented components. Blue-filled areas correspond to the PO_2_ stretching of DNA, orange-filled areas correspond to the ν(CH_3_) stretching of keratin and the purple area is not identified (A–E). Integrated area for each component across different categories (F). Lipid content estimation based on AUC calculations (G).*

*Table S1: Cosine similarity scores between attention scores averaged across all pairs of folds. For each fold the attention score is the mean of out-of-fold samples attention scores.*

| **Model/Class** | **LOB-T** | **EPI-T** | **EPI-H** | **DER-H** | **TUM-T** |
| --- | --- | --- | --- | --- | --- |
| **BCC** | 0.86 | 0.92 | 0.93 | 0.83 | -- |
| **BCC + SCC** | 0.88 | 0.90 | 0.93 | 0.86 | 0.84 |

Table S2: The AUCs of the different models for the discrimination of the targets for BCC and SCC

| **Model** | **BCC** | **BCC + SCC** |
| --- | --- | --- |
| **CNN** | 0.95 | 0.93 |
| **SVC** | 0.94 | 0.91 |
| **KNN** | 0.91 | 0.90 |
| **XGBoost** | 0.94 | 0.90 |
| **MLP** | 0.94 | 0.92 |

Table S3: key performance metrics for each individual class under the one-vs-rest binary setup. A. BCC model. B. BCC+SCC model.

**A.**

| **Class** | **AUC** | **Recall** | **Specificity** | **Precision** | **Accuracy** |
| --- | --- | --- | --- | --- | --- |
| **LOB-T** | 0.98 | 0.91 | 0.94 | 0.88 | 0.93 |
| **EPI-T** | 0.94 | 0.69 | 0.96 | 0.83 | 0.89 |
| **EPI-H** | 0.92 | 0.80 | 0.90 | 0.69 | 0.87 |
| **DER-H** | 0.96 | 0.82 | 0.96 | 0.85 | 0.93 |

**B.**

| **Class** | **AUC** | **Recall** | **Specificity** | **Precision** | **Accuracy** |
| --- | --- | --- | --- | --- | --- |
| **LOB-T** | 0.96 | 0.88 | 0.93 | 0.82 | 0.92 |
| **EPI-T** | 0.88 | 0.57 | 0.91 | 0.60 | 0.85 |
| **EPI-H** | 0.92 | 0.76 | 0.89 | 0.69 | 0.86 |
| **DER-H** | 0.97 | 0.82 | 0.97 | 0.84 | 0.95 |
| **TUM-T** | 0.86 | 0.47 | 0.93 | 0.56 | 0.86 |

1.
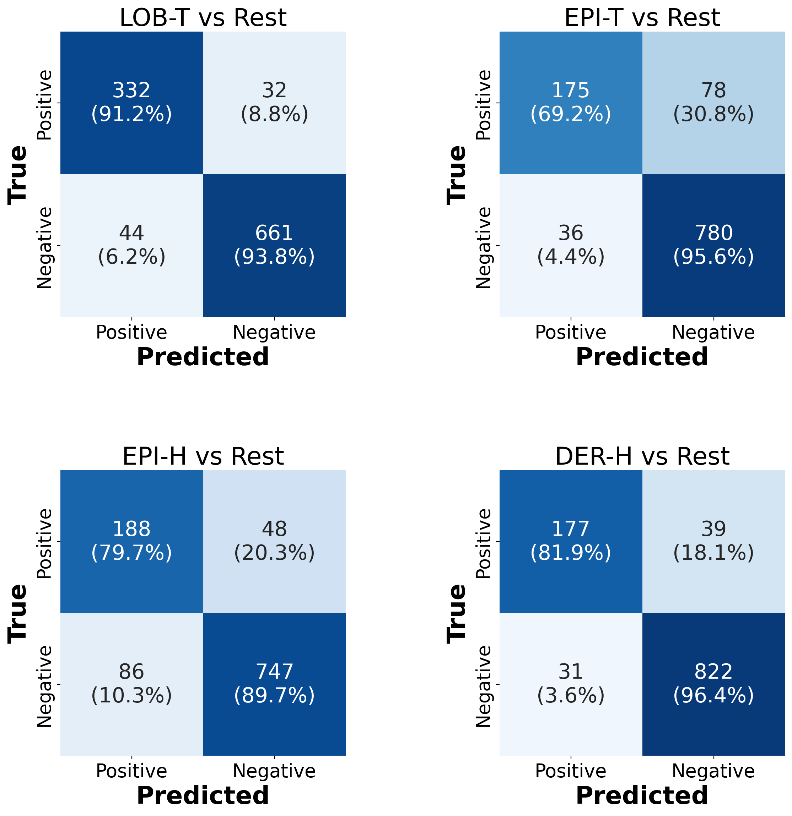

2.
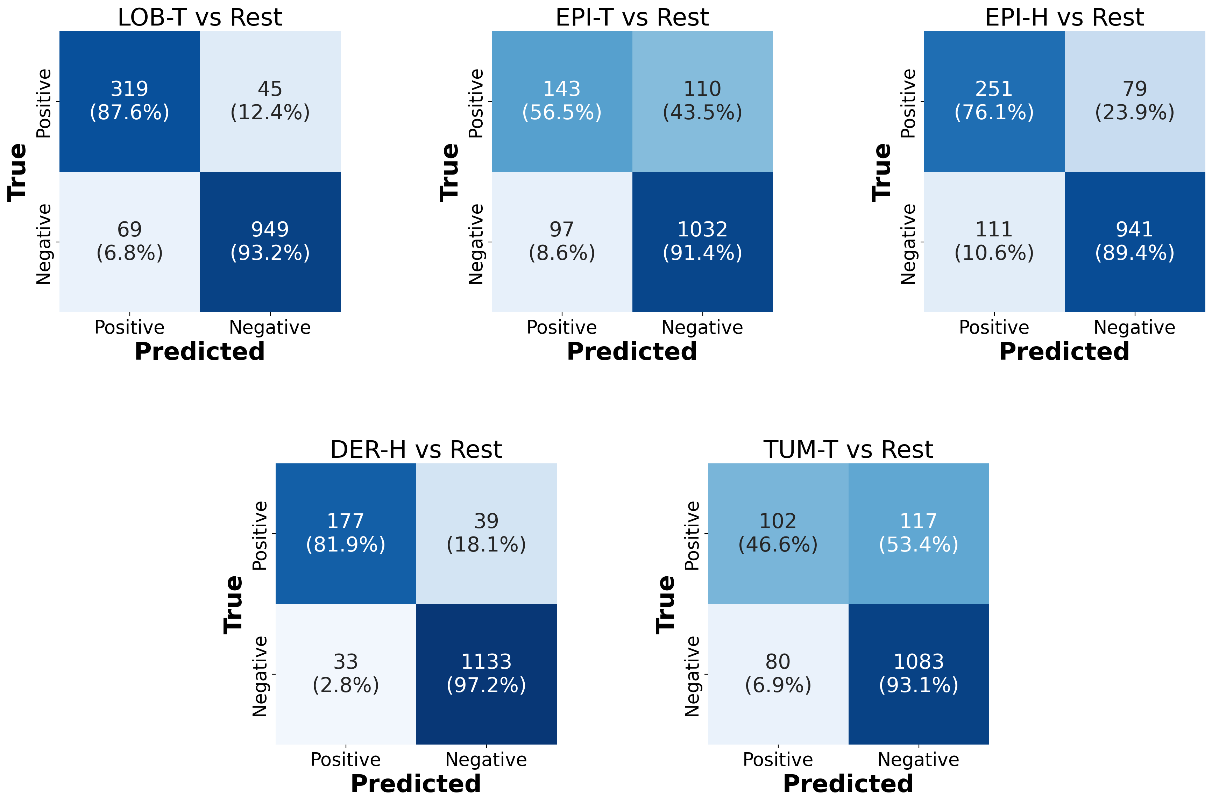


Figure S3: Individual confusion matrices with standard performance metrics (TP, FP, TN, FN), A. BCC model. B. BCC+SCC model.

Table S4: Biochemical assignments of the average spectrum based on literature. ν: stretching, ν_s_: symmetric stretching, ν_as_: antisymmetric stretching, δ: bending, τ: twisting

| **Raman shift (cm^-1^)** | **Group and vibrational mode** | **Biochemical assignments** | **References** |
| --- | --- | --- | --- |
| 821 | ν(C-C) | Proline and hydroxyproline  (collagen) | [71], [72] |
| 838 | ν(C-C) Fermi doublet | Tyrosine | [84] |
| 853 | ν(C-C) | Proline, Hydroxyproline (collagen) | [71], [66] |
|  | ν_as_(C-O-S) | Glycosaminoglycans | [71] |
|  | δ(C-H) out of plane phenyl  ν(C-C) Fermi doublet | Phenylalanine (collagen)  Tyrosine | [85]  [84] |
| 891 | ν(C-C) | Proline, Hydroxyproline (collagen) | [71] |
| 933 | ν(C-C) | Proline, Hydroxyproline | [71], [86] |
|  |  | (collagen) |  |
| 971 | ν(O-P-O) | Cellular nucleic acids | [71] |
|  | ν(C-C) | Keratin (β-sheets) | [87] |
| 1001 | ν_s_(C-C) Ring breathing | Phenylalanine (collagen) | [68], [71], [73], [74] |
| 1035 | ν(C-C) | Arginine (collagen) | [85] |
| 1042 | ν(C-C) Fermi doublet | Tyrosine | [71] |
| 1076 | ν(C-C) *gauche* | Lipid Backbones | [71], [87], [88] |
| 1092 – 1109 | ν_s_(O-P-O) | DNA backbones (nucleus) | [66], [47] |
| 1115 | δ(CH_2_) *in-plane* | Lipid Backbones | [71] |
| 1122 | ν_s_(C-C) *all trans* | Lipid Backbones | [71] |
| 1188 - 1194 | ν(C-N) | C, G, A | [89] |
|  | ν_as_(O-P-O) | Lipid Backbones | [71] |
| 1217 | ν(C-N) | Proteins | [71] |
| 1255 - 1262 | ν(C-N), δ(N-H) | Amide III Proteins  T, A, C (DNA, RNA) | [71], [90], [91] |
| 1309 | τ(CH_2_), τ(CH_3_) | Lipids, Collagen | [71] |
| 1366 | δ_s_(CH_3_) | Phospholipids | [71] |
| 1379 | δ_s_(CH_3_) | Lipids  Melanin | [71]  [67], [47], [92] |
| 1385 | δ_s_(CH_3_) | RNA (A, G, C) | [89] |
| 1394 | δ_s_(CH_3_) | Proteins | [90] |
| 1450 | δ_scissoring_ (CH) | Proteins (collagen), lipids | [49], [48], [75], [76], [77], [78] |
| 1510 | Ring breathing | DNA bases | [71] |
| 1519 | ν(C=C) | Carotenoid | [88], [71], [93] |
| 1573 | δ(N-H), ν(C-N) | Nucleic acids  DNA | [71], [79], [80], [25] |
| 1656 | ν(C=O) | Amide I (α-helix), Collagen | [71], [87], [74], [62], [94] |
| 1670 | ν(C=O) | Amide I (β-sheets) | [71] |
| 1679 | ν(C=O) | Amide I (Bound Free NADH and random coils) | [71] |
| 1734 | ν(C=O) | Esters | [88] |
| 2860 | ν_s_(CH_2_) | Lipids | [69], [70], [71] |
| 2880 | ν_as_(CH_2_) | Mostly lipids | [69], [70], [71] |
| 2908 | ν_as_(CH_2_) | Lipids | [95] |
| 2930 | ν_s_(CH_3_) | Mostly keratin | [69], [70] |
| 2955 | ν_as_(CH_3_) | Keratin | [69], [70] |
